# Planning for community well-being: Prioritizing and identifying local neighbourhood attributes of belonging.

**DOI:** 10.23889/ijpds.v7i3.2060

**Published:** 2022-08-25

**Authors:** Sarah Mah, Lori Diemert, Scott McKean, Sarah Collier, Laura Rosella

**Affiliations:** 1 Dalla Lana School of Public Health; 2 City of Toronto; 3 Toronto Public Health

## Objectives

Local neighbourhoods have great potential to foster community belonging which can improve health and well-being. In partnership with the City of Toronto, we prioritize and assess the importance of physical and social environmental attributes to community belonging. Using new data linkages, we contribute to Toronto’s community safety and well-being plan, SafeTO.

## Approach

We leverage an individual-level record linkage of respondents from multiple cycles of the Canadian Community Health Survey (CCHS, 2000 to 2017), the Canadian Vital Statistics Death Database (CVSD), and the Discharge Abstract Database (DAD) from the Centre for Population Health Data at Statistics Canada. Environmental data sources include the Census, administrative data, and open-source data. Using postal code information from the CCHS, we connected each respondent with their neighbourhood’s attributes, which include but are not limited to proximity to amenities (such as childcare, libraries and public transit), the Canadian Active Living Environment measure, green space, and air quality.

## Results

Of the 74,000 CCHS respondents from the Toronto census metropolitan area (representing a population of 4 to 5 million annually), an overall rate of 86% agreed to link and share their data, which renders an estimated linked sample of approximately 63,600 respondents. Across the entire linkage, 54% of respondents are linked to a record in the DAD between 1999/00 and 2017/18, and 10% are linked to a death record in the CVSD between 2000 and 2017. In consultation with municipal agencies and community stakeholders, we will prioritize the environmental attributes that are most relevant to community belonging and well-being, and comprehensively model the relationship between these attributes, community belonging and downstream health outcomes using multivariable regression and time-to-event models.

## Conclusion

This on-going work contributes to the development of innovative approaches for using multi-sector data to inform decision making, as per the goals of SafeTO. Results from the analyses will be used to identify environmental attributes potentially important for community belonging and will support municipal city planning and resource allocation.

